# Artificial intelligence-based volumetric measurements for longitudinal clinical assessment of treatment response in high-grade gliomas: Validation across institutional and public datasets

**DOI:** 10.1093/noajnl/vdag045

**Published:** 2026-02-17

**Authors:** Zerubabbel K Asfaw, Tirone Young, Gianina Hernandez Marquez, Cole Brown, Lewis E Tomalin, Puneet Belani, Amish Doshi, Isabelle M Germano

**Affiliations:** Department of Neurosurgery, Icahn School of Medicine at Mount Sinai, New York City; Department of Neurosurgery, Icahn School of Medicine at Mount Sinai, New York City; Department of Surgery, University of Alabama at Birmingham, Birmingham; Department of Neurosurgery, Icahn School of Medicine at Mount Sinai, New York City; Department of Population Health Science and Policy, Icahn School of Medicine at Mount Sinai, New York City; Department of Radiology, Icahn School of Medicine at Mount Sinai, New York City; Department of Radiology, Icahn School of Medicine at Mount Sinai, New York City; Department of Neurosurgery, Icahn School of Medicine at Mount Sinai, New York City

**Keywords:** artifical intelligence, high grade gliomas, RANO, treatment response, volumetric measurement

## Abstract

**Background:**

High-grade gliomas (HGGs) require ongoing imaging to guide treatment, traditionally relying on labor-intensive and variable manual MRI measurements. While FDA-cleared artificial intelligence (AI) tools offer automated tumor volume segmentation, their clinical utility in decision-making remains understudied. This study assesses the utility and limitations of an FDA-cleared AI-based tool across public and institutional datasets, comparing its output with multidisciplinary tumor board (MDTB) assessments.

**Methods:**

We applied the FDA-cleared, AI-based tool Neosoma HGG to quantify tumor volumes in 214 subjects from public datasets and 49 from an institutional cohort. AI-derived volumes were compared to expert manual and other AI-based measurements. Therapeutic response assessments using RANO criteria were evaluated against MDTB diagnoses. Segmentation times were analyzed using mixed-model regression.

**Results:**

We analyzed 1648 MRI sequences of 95 HGG patients across three datasets. Contrast-enhancing (CE) tumor volumes were consistent across AI platforms, and Neosoma HGG significantly reduced segmentation time (pre-operative: 210.5s, post-operative: 179s vs. 15 s, *P* < .0001). AI-informed disease state assessments showed an overall moderate agreement with MDTB diagnoses for progressive disease (*k* = 0.45, *P* < .00001). Key discrepancies arose from limitation of Neosoma HGG in distinguishing pseudo-progression from tumor progression. T2-FLAIR-derived volumes varied significantly between AI platforms (*P* < .001), with discordances largely due to over-segmentation beyond the tumor region.

**Conclusion:**

AI-based volumetric segmentation has the potential to improve efficiency and standardization in monitoring HGG, especially for CE tumor burden. However, moderate concordance with MDTB assessments and difficulties with FLAIR imaging underscore its current limitations. AI should serve as a clinical decision support tool, with further refinement needed to improve specificity and integrate multimodal imaging data.

Key Points

- AI-based volumetric segmentation for HGG offers accurate and efficient analysis, significantly reducing processing time while maintaining strong alignment with expert measurements.
- Moderate agreement with MDTB assessments supports its use as an adjunct tool for evaluating treatment response.

Importance of the Study

This study demonstrates the clinical utility and feasibility of integrating an FDA-cleared AI platform into the longitudinal management of high-grade gliomas (HGG). Neosoma HGG showed strong concordance with expert-derived tumor measurements, significantly reduced processing time compared to manual methods, and achieved moderate to substantial agreement with disease state assessments by the multidisciplinary tumor board (MDTB). While AI has the potential to increase efficiency, objectivity, and consistency of applying RANO criteria, it is not a replacement for expert clinical judgment—particularly in complex cases. Current limitations include challenges in interpreting fluid attenuated inversion recovery (FLAIR) signal changes and differentiating treatment-related effects (pseudo-progression) from true progression. Despite these, the findings support the growing role of AI as a complementary tool in neuro-oncology. Future research is needed to refine its diagnostic specificity and validate its performance across broader, prospective patient populations.

## Introduction

High-grade gliomas (HGG), including glioblastoma (GB), are the most common and aggressive primary brain tumors in adults, comprising over 50% of all malignant primary brain tumors.^1^ In the United States, the incidence of primary central nervous system (CNS) tumors is 24.83 per 100 000 population, with gliomas accounting for 26.3% of these cases. GB, in particular, has an incidence of 2 to 3 per 100 000 and is more prevalent in men.^2^ The prognosis remains poor, with a median overall survival of approximately 12 to 15 months and a 5-year survival rate of only 6.8%.^1^^,^^3^ Despite aggressive treatment, including maximal surgical resection followed by combined radiation therapy and chemotherapy, tumor recurrence occurs in about 98% of patients due to the infiltrative nature of the disease.^4-7^ Moreover, the financial burden is substantial, with GB representing one of the most expensive cancers to treat, largely because of the high-intensity resource utilization.^8-10^

Given the high recurrence rate and poor prognosis associated with HGG, consistent and accurate longitudinal monitoring of treatment response is essential.^11^ Magnetic resonance imaging (MRI) remains the cornerstone of post-treatment surveillance, with radiographic evaluations guided by standardized criteria such as the Response Assessment in Neuro-Oncology (RANO), its updated counterpart, the modified RANO (mRANO), and the most recent RANO 2.0.^11-13^ However, widespread implementation of uniform monitoring practices remains a challenge. One major barrier is the lack of consensus on the optimal method for assessing tumor size changes—whether traditional bidimensional diameter measurements or more comprehensive three-dimensional volumetric assessments offer greater accuracy.^14^^,^^15^ While volumetric segmentation is often viewed as the more precise approach, performing it manually is labor-intensive, time-consuming, and susceptible to interrater variability.^16^^,^^17^ These limitations hinder its routine use, especially in busy clinical settings where efficiency is critical.^17^

Recent advancements in artificial intelligence (AI) and deep learning have led to the development of automated tumor segmentation tools aimed at addressing these limitations.^17-19^ Among these, the Neosoma HGG software, an FDA-cleared AI-based solution, performs rapid, repeatable volumetric analysis using a limited MRI sequence set within a five-sub model architecture that generates consensus outputs via majority voting.^17^

This study aims to (1) compare the accuracy of volumetric and bidimensional measurements obtained via Neosoma HGG with expert-derived measurements in tracking longitudinal tumor changes; (2) evaluate how Neosoma HGG volumetric assessments stack up against those obtained through manual segmentation and other AI-based tools; and (3) determine whether disease states informed by Neosoma HGG align with diagnoses made by multidisciplinary tumor board (MDTB) based on standard clinical data and RANO criteria. Through these aims, the study attempts to clarify the potential of AI-based tools to enhance consistency, accuracy, and efficiency in neuro-oncologic care.

## Methods

### Study Cohorts and Eligibility Criteria

The study was based on three distinct patient cohorts. The first two cohorts were acquired from publicly available online datasets: the RTOG 0625/ACRIN 667 (ACRIN) study and the LUMIERE study.^20^^,^^21^ The ACRIN dataset consisted of the baseline and longitudinal follow-up MRI of 123 subjects with recurrent GB who participated in a multi-center, randomized, phase II trial of bevacizumab with irinotecan or temozolomide.^20^ MRIs were obtained at baseline, week 2, and every 2 cycles of treatment. This dataset featured expert-generated manual bidimensional and volumetric tumor measurements. The LUMIERE dataset comprised longitudinal MRI dataset from 91 subjects with GB treated under the standard Stupp protocol.^21^^,^^22^ It included 638 automated tumor segmentations generated by the DeepBraTumIA software, an AI-automated segmentation tool without FDA clearance. MRIs were obtained at baseline, week 1-2, week 15-16, week 30-35, week 60-65, week 80 or later.

The third cohort consisted of 49 subjects who received neurosurgical oncology treatment at the Mount Sinai Health System between January 1, 2018, and December 31, 2022. Eligible participants were adults (≥18 years) with histologically confirmed high-grade glioma (WHO Grade 3 or 4) who underwent a preoperative MRI (TP0) followed by imaging at four standardized postoperative time points—baseline/within 72 hours after surgery (TPB), and three subsequent imaging time points labeled TP1 (15-16 weeks), TP2 (30-35 weeks), and TP3 (week 60 or greater). The MRI-related inclusion criteria were (1) availability of T1 weighted (T1W), T1 weighted with contrast (T1W-CE), T2 weighted (T2W), and T2 Fluid-Attenuated Inversion Recovery (T2-FLAIR) sequences, (2) parameters set per standardized Brain Tumor Imaging Protocol (BTIP),^23^ and (3) absence of motion artifacts.

In total, the study involved 263 subjects, each with five MRI time points and four imaging sequences per scan, yielding a potential dataset of 5260 individual MRI assessments. Although all subjects had a confirmed GB diagnosis, some possessed a mutant IDH profile and received the same treatment as those with wild-type IDH, as their diagnoses preceded the inclusion of IDH status in GB diagnosis.^24^^,^^25^ This study was approved by the Institutional Review Board of the Icahn School of Medicine at Mount Sinai (IRB #22-01444) and adheres to the Strengthening the Reporting of Observational Studies in Epidemiology (STROBE) guidelines.

### Data Collection and Analysis

The following data was extracted from the ACRIN study: demographic and clinical characteristics, tumor diameter and volume across longitudinal time points derived from T1W-CE, and T2-FLAIR sequences. The LUMIERE dataset similarly provided demographic and clinical data, along with tumor volume and necrotic core measurements from T1W-CE, and from T2-FLAIR imaging.

For the institutional cohort, data were obtained from the electronic medical records, including patient age, gender, and race. Additional clinical variables collected included presenting symptoms, lesion laterality, extent of resection (EOR), presence of new measurable contrast-enhancing lesions, neurological status, steroid use and any change in steroid dosage compared to baseline, date of last follow-up, and date of death. Disease state, as determined by weekly MDTB discussions. During the meeting, each patient’s images are reviewed with input from the neuroradiology team providing measurements and from the neuro-oncology team providing clinical information necessary to reach a response score based on RANO 2.0 criteria was also extracted and compared to the disease status generated by the Neosoma HGG software, as described below.^13^ Disease states were categorized in accordance with RANO 2.0 criteria as complete response (CR), partial response (PR), progressive disease (PD), or stable disease (SD).^13^ Discrepancy between MDTB and Neosoma-informed disease sate were further analyzed by the authors. Given the retrospective nature of our study, the authors had access to follow-up imaging and clinical data beyond the time point of the initial MDTB adjudication. This longitudinal information allowed the authors to retrospectively determine whether the initial apparent progression was, in fact, pseudo-progression—characterized by transient radiographic worsening followed by stabilization or improvement without a change in therapy. Thus, adjudications of pseudo-progression were informed by subsequent outcomes, which would not have been available in a real-time clinical setting.

### Imaging Analysis

**Figure 1.**
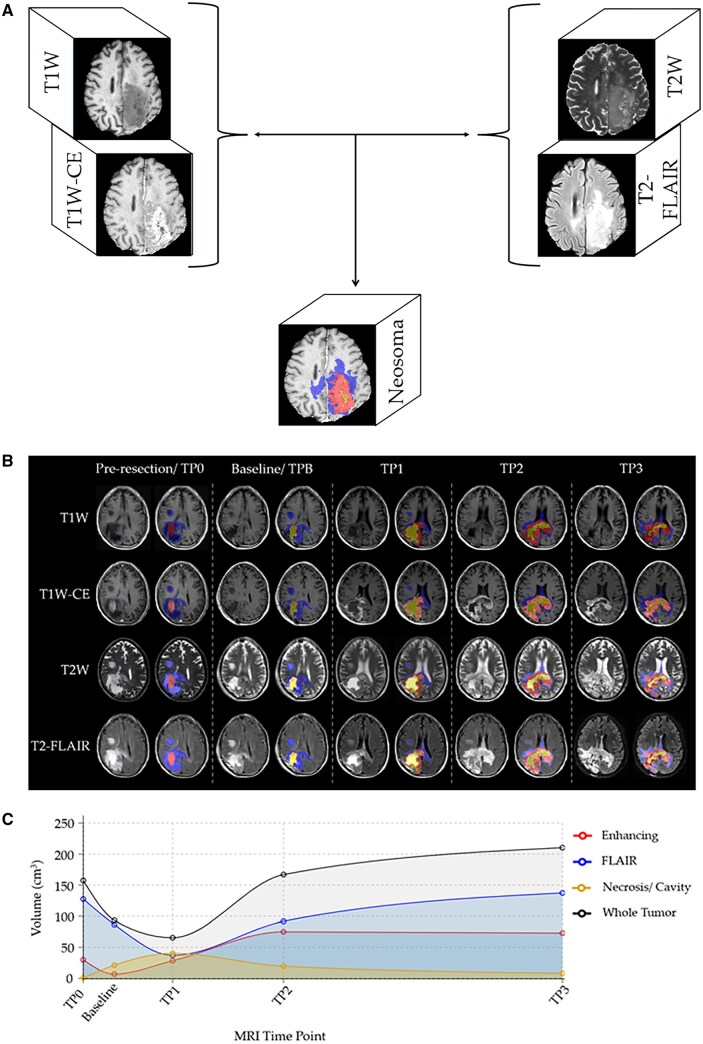
(A) Diagram demonstrating how the Neosoma HGG software produces volumetric measurements and final visualization. (B) Case example of Neosoma HGG segmentation on axial MR images of a subject with HGG at each of the five studied time points. (C) Volumetric measurement of each tumor component across all study time points. The MR sequences include T1W= T1 weighted, T1W-CE= T1 weighted with contrast, T2W= T2 weighted, and FLAIR= Fluid-Attenuated Inversion Recovery. Neosoma HGG highlights T2-FLAIR increased signal (blue), contrast-enhanced tumor (red), and the necrotic tumor cavity (yellow).

All MRI scans were uploaded to the Neosoma HGG server, where automated preprocessing—including skull-stripping—and quality control were performed prior to analysis. Quality control for each data set consisted of ensuring the set contained imaging parameters as previously published,^17^ summarized by the authors in their Table 2. There is no manual quality control needed for this platform. If one or more of the 4 key sequences are missing, the platform will not provide the data. After loading the images, if artifacts (ie motion, metallic, etc.) is identified, the clinical team/investigator might decide to exclude. the images were further screened to ensure no lack of motion and/or omissions and/or technical issue.^17^ Eligible data set were then processed to segment T1W-CE, necrotic, T2-FLAIR (Figure 1A and B). The MRI obtained at TPB served as the baseline for comparison relative to the subsequent timepoints (Figure 1C). The time for acquiring such measurements was recorded to compare with that of manual measurement alternatives.

**Table 2.** Evaluation of diagnostic discrepancies between the multi-disciplinary tumor board (MDTB) and the Neosoma HGG AI-informed approach in assessing progressive vs. non-progressive disease. An independent expert adjudicated the correct diagnosis based on a review of imaging, AI-driven volumetric segmentation, and the patient’s clinical status.

Reason for discrepancy	Count (*n* = 29)	Correct diagnosis provided by
Post-operative changes	5 (17%)	Multidisciplinary tumor board
Inability to differentiate between pseudo-progression and progression	6 (21%)	
Hyper-sensitive FLAIR rendering by AI-automated segmentation	7 (24%)	
Multifocal disease	4 (14%)	Neosoma HGG-informed assessment
Contrast Enhancing disease volume close to the cutoff of ≥25% increase	7 (24%)	

All MRI sequences from the ACRIN and LUMIERE datasets were processed using the Neosoma HGG software to generate both bidimensional product and volumetric tumor measurements. For the ACRIN dataset, Neosoma-derived measurements were compared against expert-generated bidimensional and volumetric values to assess concordance. In the LUMIERE dataset, tumor volumes produced by Neosoma HGG were compared with those generated by DeepBraTumIA, Images showing a volume difference >10% between Neosoma and the comparator platform were reviewed by two authors (IMG/PB). These images were presented side-by-side with their respective derived contours visible. Each investigator independently selected the segmentation they judged to be the most accurate.

The MRI acquired at TP0 and TPB time points, for the institutional cohort, were used for manual volumetric segmentation to determine HGG lesion volume via Vitrea (version 7.8, Canon Medical Systems, Australia), a software commonly available to neuroradiologists. Three investigators (ZKA, TY, and CB) received equal training from a fully trained neuro-radiologist on how to use Vitrea for manual segmentation. The following variables were recorded for each T1W-CE MR images analyzed with Vitrea: lesion volume, transverse diameter, axial diameter, and the total time spent analyzing the imaging. Although uploading MR images from the institutional Picture Archiving and Communication System (PACS) was time-consuming, total analysis time was recorded only after the images were fully loaded onto the Vitrea server, ensuring representative results for institutions using similar software but different PACS setups.

### Statistical Analysis

Continuous variables were summarized as means ± standard deviation; categorical variables were expressed as percentages. Inter-rater reliability for manual segmentations was assessed using intraclass correlation coefficients (ICC). A mixed-model regression was used to compare segmentation times across investigators and between Vitrea and Neosoma HGG.

For public datasets, Wilcoxon Signed Rank Tests assessed statistical differences in percent change between manual or DeepBraTumIA measurements and Neosoma HGG-derived estimates. For institutional cases, Cohen’s kappa (κ) was calculated to assess agreement between MDTB-assigned and AI-inferred disease states. Interrater reliability were interpreted as follows: values = 0 as indicating no agreement and 0.01-0.20 as none to slight, 0.21-0.40 as fair, 0.41-0.60 as moderate, 0.61-0.80 as substantial, and 0.81-1.00 as almost perfect agreement, as published.^26^ Owing to MRI exclusions by Neosoma HGG for quality assurance and the limited representation of subjects in CR and PR categories, disease states were consolidated into progressive disease (PD) and non-progressive disease (non-PD) prior to comparison, emphasizing the key clinical decision point of determining disease progression. Expert adjudication (IMG, PB) was performed to evaluate cases with discordant classifications. A *P*-value <.05 was considered statistically significant. All statistical analyses were performed using Microsoft Excel (Seattle, WA) and RStudio (version 2024.04.1).

## Results

### Cohort Demographics and Clinical Characteristics

From the public datasets, 22 subjects from the ACRIN and 24 subjects from the LUMIERE datasets met all eligibility criteria for inclusion. Demographics and clinical variables for all subjects, including the 49 patients from the institutional cohort, are summarized in Table 1. The mean age of subjects in ACRIN (51 ± 12 years) was less than that in the LUMIERE (63 ± 11 years) and institutional cohorts (67 ± 13 years). There was similar representation of female subjects across all cohorts (42%-45%). While all patients in the ACRIN cohort identified as White, 55% of the institutional cohort were White subjects. The most common presenting symptom for subjects in the institutional cohort was neurological deficit (47%), followed by headache (27%). The majority of subjects in the LUMIERE cohort (75%) and all subjects in the institutional cohort had an IDH wild-type molecular profile. The IDH status was not reported for the ACRIN cohort. In our Institutional cohort, gross total resection was achieved in 63% of patients, and 51% were discharged home postoperatively (Table 1).

**Table 1.** Demographic and clinical characteristics of all cohorts included in the study.

	ACRIN cohort^19^	LUMIERE cohort^20^	Institutional cohort
Variable	Count or Mean (*n* = 22)	Count or Mean (*n* = 24)	Count or Mean (*n* = 49)
Age	51 ± 12	63 ± 11	67 ± 13
Gender (Female)	10 (45%)	10 (42%)	21 (43%)
Race/Ethnicity			
Black	0	NA	6 (12%)
Hispanic/Latinx	0	NA	8 (16%)
White	22 (100%)	NA	27 (55%)
Other	0	NA	8 (16%)
Presenting symptom			
Altered mental status	NA	NA	2 (4%)
Headache	NA	NA	13 (27%)
Loss of consciousness	NA	NA	1 (2%)
Neurological deficit	NA	NA	23 (47%)
Seizures	NA	NA	6 (12%)
Stable	NA	NA	4 (8%)
Tumor location			
Frontal	3 (14)	NA	22 (45%)
Temporal	10 (45)	NA	15 (31%)
Parietal	4 (18)	NA	4 (8%)
Occipital	5 (23)	NA	2 (4%)
Multilobed	4 (18)	NA	6 (12%)
IDH status			
Wild type	NA	18 (75%)	49 (100%)
Mutant	NA	1 (4%)	0
Negative^a^	NA	2 (8%)	0
Unavailable	NA	3 (13%)	0
MGMT promoter status			
Methylated	NA	10 (42%)	15 (31%)
Unmethylated	NA	11 (46%)	15 (31%)
Unavailable/Indeterminate	NA	3 (12%)	19 (39%)
Extent of resection			
Gross total resection	NA	NA	31 (63%)
Sub total resection	NA	NA	18 (37%)
Disposition after resection			
Acute Rehab	NA	NA	21 (43%)
Assisted living facility	NA	NA	1 (2%)
Home	NA	NA	25 (51%)
Sub-acute rehab	NA	NA	2 (4%)

a IDH immunohistochemistry was negative for subjects where sequencing would have been necessary for a definite result.

### Bidimensional and Volumetric Tumor Measurements Assessment

We analyzed 1,648 MRI sequences of 95 HGG patients across all datasets. From the ACRIN dataset, 352 MRI sequences from 22 subjects were available for comparison. There was no statistically significant difference in both the bidimensional product and volumetric measurements between expert-derived manual measurements and those derived from Neosoma HGG (*P* > .05). For the LUMIERE database, 565 MRI sequences from 24 were included in the analysis. There was no significant difference in tumor burden volume measurements based on T1W-CE comparing DeepBraTumIA and Neosoma HGG-derived measurements (*P* = .09). When comparing volumes in T2-FLAIR sequences, a statistically significant higher volume obtained from Neosoma HGG as compared to DeepBraTumIA (*P* < .001) was found. A blinded, random expert comparison of the 56 T2-FLAIR images with over a 10% difference in volume measured by Neosoma HGG and DeepBraTumIA revealed that DeepBraTumIA provided a slightly more accurate T2-FLAIR volume segmentation in 33/56 (59%) images, albeit not statistically significant. An in-depth analysis of discrepant images demonstrated that ischemic changes and white matter changes occurring outside the tumor bed were recognized and detected more often by Neosoma HGG than DeepBraTumIA.

### Volumetric Segmentation: Accuracy and Efficiency Comparison

**Figure 2.**
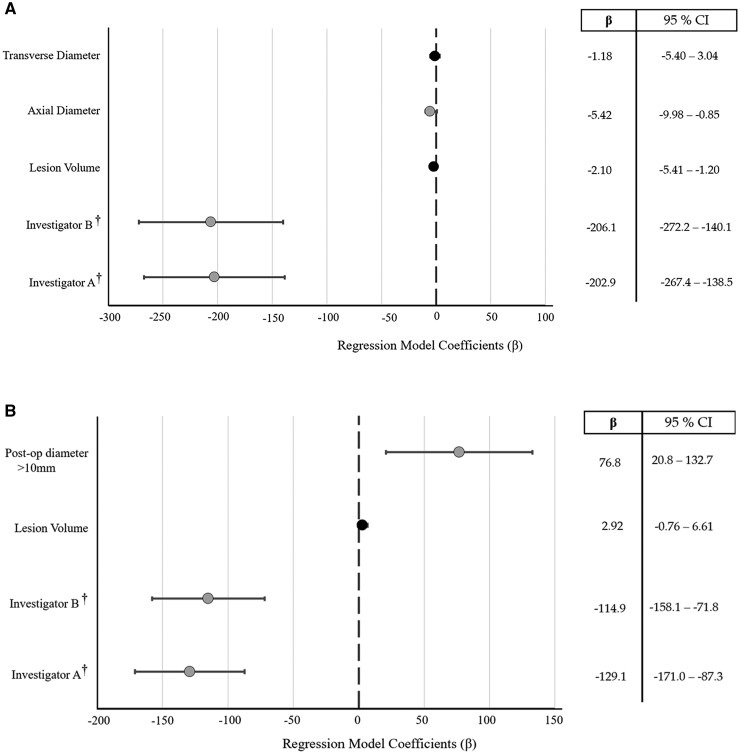
Forest plot illustrating that lesion size and investigator identity significantly affect the time required for manual volumetric segmentation of HGG on (A) pre-operative and (B) post-operative imaging. “*” Gray circles indicate statistically significant predictor variables (*P* < .05). “†” denotes that Investigator C serves as the reference group in the model.

For the 49 subjects in the institutional cohort, 57 contrast-enhancing (CE) lesions were manually segmented at TP0 and TPB. Neosoma HGG significantly reduced the time required for volumetric segmentation compared to manual segmentation via Vitrea (pre-operative: 210.5 s, post-operative: 179 s vs. 15 s, *P* < .0001) (Figure S1). Time required for manual segmentation varied significantly by investigator (*P* < .0001) and tumor axial diameter (*P* = .021). Larger axial diameters were associated with reduced analysis time, while transverse diameter and overall volume had no impact (Figure 2A).

Postoperative imaging analysis revealed consistent trends, including significant investigator-dependent variability in segmentation time (*P* < .0001) and a positive association between segmentation time and residual tumor size (*P* = .0075), with larger post-operative tumor volumes requiring more time to segment (Figure 2B). Inter-rater reliability for manual segmentation was good for pre-operative lesions (ICC = 0.67; 95% CI: 0.55-0.78) and moderate postoperatively (ICC = 0.53; 95% CI: 0.38-0.67), both statistically significant (*P* < .0001). Regression analysis revealed no significant variation in lesion volume estimates across investigators after controlling lesion size, subject identity, and analysis time.

### Disease State Assessment: Concordance between MDTB and AI-Derived Results

Out of 245 MRI scans across longitudinal time points, 183 (75%)—33 pre-operative and 150 post-operative scans—met Neosoma HGG AI-derived quality control standards. Of the 62 scans excluded, 90% were missing either T2W or FLAIR sequences. The remaining scans were excluded due to the absence of a T1W-CE sequence for medical reasons or due to significant artifacts (ie motion, metallic, other) identified after the images were loaded to the platform. Notably, volumetric acquisitions were not required, and therefore no scans were excluded on that basis.

AI-informed disease states were determined for 101 scans at TP1, TP2, and TP3. AI-informed disease states were determined for 101 scans at TP1-TP3, while 49 scans obtained during the earliest post-operative period served as the baseline reference per RANO criteria. The highest category-specific agreement between Neosoma HGG and MDTB assessments was observed for PD: TP1 (75%), TP2 (68%), and TP3 (37.5%). The agreement for SD was lower: TP1 (35.7%), TP2 (26.7%), and TP3 (14.3%). No agreement was recorded for PR, but full agreement was seen in one patient with a complete response (CR) at TP1 and TP2 (Table S1).

**Figure 3.**
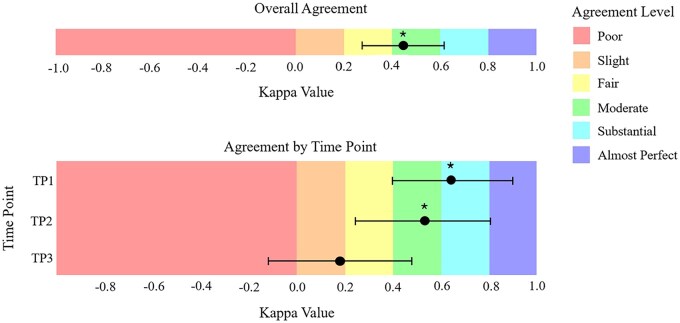
Agreement between the multidisciplinary tumor board (MDTB) and the Neosoma HGG-informed approach in classifying PD vs. non-PD according to RANO criteria. Each panel displays the overall Cohen’s kappa across all time points and the kappa values for individual time points. “*” indicates statistically significant agreement (*P* < .05).

After consolidating disease states into two categories, PD and non-PD, Cohen’s kappa analysis was used to determine the disease state determined by MDTB versus Neosoma HGG-informed approach. There was a significant moderate agreement (*k* = 0.45, 95% CI [0.28, 0.62], *P* < .00001) in the identification of PD by MDTB compared to the Neosoma HGG-informed approach for the overall cohort. An analysis for each time point revealed that early time points had a more robust statistically significant agreement than the latest time points. There was a significant substantial agreement at TP1 (*k* = 0.64, 95% CI [0.38, 0.90], *P* < .0001) and a significantly moderate agreement at TP2 (*k* = 0.53, 95% CI [0.24, 0.81], *P* < .001- for Cohen’s agreement scale refer to methods). However, the degree of agreement was not significant at TP3 (*k* = 0.18, 95% CI [−0.12, 0.48], *P* = .19) (Figure 3).

**Figure 4.**
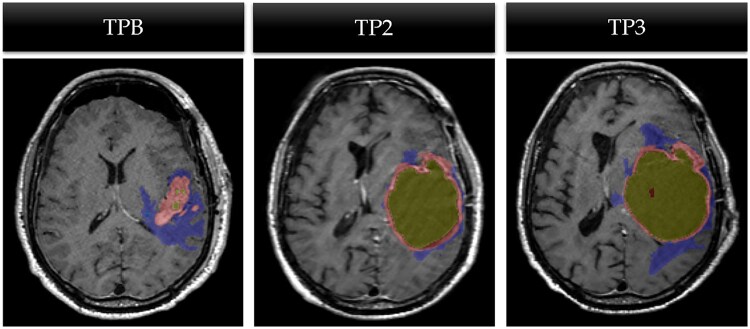
Case example of Neosoma HGG segmentation on axial MR images of a subject with increased resection cavity volume due to accumulation of cerebrospinal fluid showing lack of cystic component at TPB (time point baseline, left panel), with cystic accumulation within the resection cavity at TP2 (time point 2, center panel) and additional cystic enlargement at TP3 (time point 3 right panel). The area of FLAIR-abnormality is shown in blue and its increment over time is most likely secondary to the effects of the increased cyst volume not the tumor recurrence.

Of the 101 analyzed timepoints, 29% exhibited discrepancies between the MDTB and the Neosoma HGG-informed approach in diagnosing PD versus non-PD. An expert-led adjudication of discrepancies revealed that the MDTB consensus had the correct diagnosis in 62% of cases, largely due to their ability to interpret imaging features suggestive of PD within a clinical context indicating pseudo-progression (24%). Additionally, the MDTB accurately identified FLAIR signal changes unrelated to actual progression, such as signals in irrelevant brain regions or increased FLAIR signal due to resection cavity expansion with cerebrospinal fluid (24%) (Figure 4). Furthermore, in 14% of discrepant cases, the MDTB correctly distinguished post-operative changes—included in AI-based volumetric segmentation—as non-contributory to disease progression. Conversely, the Neosoma AI-informed approach led to a correct diagnosis in 38% of cases, primarily through precise assessment of enhancing disease near the ≥25% in sum of products or perpendicular diameters or >40% increase in total volume of enhancing lesion increase threshold for progressive disease (21%) and quantification of multifocal disease volume (17%) (Table 2).

## Discussion

HGG are the most common and aggressive primary brain malignancies, characterized by a poor prognosis and a five-year survival rate of less than 7%.^27^^,^^28^ Accurate and timely assessment of disease progression is critical for ­guiding treatment, evaluating therapeutic response, and determining clinical trial eligibility.^18^^,^^29^^,^^30^ Serial MRI-based assessments—anchored in standardized frameworks such as the RANO and modified RANO criteria—remain the backbone of longitudinal disease monitoring.^11^^,^^12^ However, challenges persist in distinguishing true progression from pseudo-progression, particularly in the early post-treatment period when transient treatment-related changes can mimic disease recurrence.^31^^,^^32^ Pseudo-progression occurs in approximately one-third of GB patients, making diagnostic accuracy during longitudinal follow-up both clinically and therapeutically consequential.^32^

A growing body of literature supports the potential of AI tools to augment neuro-oncologic imaging interpretation.^33^^,^^34^ These tools offer rapid and reproducible volumetric measurements that reduce the time burden and variability associated with manual segmentation.^34^^,^^35^ The results of this study highlight three key findings. First, Neosoma HGG produced volumetric measurements that were highly concordant with expert-derived values from the ACRIN dataset, validating its accuracy for both bidimensional and volumetric assessments. This finding is consistent with previous studies demonstrating comparable extent of resection and residual tumor volume measurements between manual and automated methods.^19^ Importantly, automated segmentation enables objective and consistent application of RANO and mRANO criteria, helping to streamline and standardize disease status assessment.

Second, Neosoma HGG demonstrated a substantial time advantage over manual segmentation, with a marked reduction in processing time, especially relevant to high-volume clinical settings. Manual segmentation, as our data and prior studies show,^17^^,^^19^^,^^34^ is time-intensive, prone to inter-rater variability—particularly in post-operative settings—and is sensitive to tumor size and morphology. The inter-rater reliability observed in our study (ICC = 0.67, 95% CI: 0.55-0.78 pre-op; 0.53, 95% CI: 0.38-0.67 post-op) further underscores the need for more standardized approaches. Automated tools like Neosoma HGG could help overcome these inconsistencies by providing reproducible, observer-independent measurements.

Third, we observed moderate to substantial agreement (κ = 0.45-0.64) between AI-informed disease state assessments and those determined by the MDTB. This agreement was strongest in identifying progressive disease, suggesting the sensitivity of Neosoma HGG in detecting clear volumetric increases or new enhancing lesions. However, discrepancies increased over time—particularly at later follow-up points—likely reflecting the complex interplay of treatment effects and true progression.^36^^,^^37^ Notably, expert adjudication revealed that 24% of discrepancies were due to pseudo-progression, highlighting current limitations of Neosoma HGG in interpreting nuanced imaging changes without clinical context. Notably, an error in the diagnosis of progression versus pseudo-progression has important clinical implications, including enrollment in clinical trials. This reinforces the importance of expert interpretation when clinical judgment can discern subtle imaging features beyond the current reach of AI.

Our findings also identified limitations in Neosoma HGG interpretation of T2-FLAIR abnormalities. Compared to DeepBraTumIA, Neosoma HGG detected a broader range of FLAIR signal abnormalities, often including areas outside the tumor bed. While potentially useful for identifying early T2-FLAIR or subtle progression, this also risks overestimating disease burden—especially in the context of postoperative resection cavities or treatment-related changes. These results are in line with prior reports and suggest that while AI can quantify changes, refinement is needed to improve specificity in post-treatment imaging.^38^

Another important consideration is the limitation of the RANO framework itself, which compares the most recent MRI to the baseline scan, potentially overlooking interval changes at intermediate time points.^12^ In our study, the longitudinal volumetric tracking of Neosoma HGG enabled the detection of subtle trends that may be missed by traditional interpretation. This suggests that AI-driven platforms could enhance the clinical application of RANO criteria, particularly in multifocal disease or near-threshold volumetric changes where visual interpretation may fall short.

Crucially, our study also explored the potential for AI to serve as a clinical decision-support tool, rather than a replacement for expert evaluation. While Neosoma HGG offers fast, objective measurements, it lacks the capacity to fully integrate complex clinical factors, such as steroid use, neurological decline, or treatment timing—elements central to MDTB decision-making. Thus, AI tools should be viewed as complementary, providing high-throughput quantitative support that allows clinicians to focus on synthesizing imaging findings within a broader clinical context.

Despite these promising results, several limitations must be acknowledged. The elimination of many subjects from both the public datasets and the institutional cohort at various stages of analysis make the results susceptible to selection bias. It also highlights the fact that specific MRI sequences are needed to allow use of AI-driven platforms. Furthermore, the platform’s stringent imaging requirements led to the exclusion of about one-fourth of the scans, which reduced statistical power and disrupted longitudinal continuity, potentially introducing selection bias. Neosoma HGG is currently cleared only for volumetric segmentation, restricting its utility in evaluating pseudo-progression or radiation necrosis. Future studies should aim to validate these findings in larger, multicenter, prospective cohorts and explore the integration of AI with advanced imaging modalities such as perfusion or diffusion MRI to improve diagnostic granularity.

## Conclusion

This study demonstrates the feasibility and clinical -applicability of integrating Neosoma HGG, an FDA-cleared AI tool. Neosoma HGG offers accurate and efficient bidimensional and volumetric measurements that can standardize radiological tumor assessment, enhance the application of treatment response criteria, and deliver substantial time savings in clinical workflows of high-volume settings. While the moderate concordance between AI-informed disease states and MDTB evaluations is encouraging, current AI capabilities should be viewed as a valuable complement—rather than a replacement—for expert clinical judgment. Further refinement and validation of these tools, particularly in complex scenarios such as distinguishing pseudo-progression from true progression, will be critical to optimizing the role of AI in neuro-oncology.

## Supplementary Material

vdag045_Supplementary_Data

## Data Availability

The data underlying this article cannot be shared publicly for the privacy of individuals who participated in the study. The data will be shared at a reasonable request by the corresponding author.

## Lay Summary

High-grade gliomas are aggressive brain tumors that require frequent MRI scans to monitor treatment, which are often reviewed manually and can vary between doctors. The authors of this study wanted to test an artificial intelligence tool called Neosoma to see how well it could measure tumor size and detect changes compared to expert review. To do this, they analyzed more than 1600 MRI scans from 95 patients. They found that Neosoma measured tumor volume quickly and pro­duced results consistent with other computer systems, although there were some differences in how it mea­sured areas of swelling around the tumor.
