# Supplementary material for: Artificial intelligence-based volumetric measurements for longitudinal clinical assessment of treatment response in high-grade gliomas: Validation across institutional and public datasets
Source: Neurooncol Adv. 2026 Feb 17;8(1):vdag045. doi: 10.1093/noajnl/vdag045 (PMC12989098; doi:10.1093/noajnl/vdag045)
Supplement: vdag045_Supplementary_Data [file vdag045_supplementary_data.zip › Supplementary Table and Figure Legends.docx]

**Table S1**: The disease state at each study time point in comparison to baseline imaging based on multidisciplinary tumor board and Neosoma HGG-informed diagnosis.

| Time Point | Disease State | Multidisciplinary Tumor Board | Neosoma HGG-informed Disease State based on RANO Criteria |
| --- | --- | --- | --- |
| P1 | CR | 1 (2%) | 1 (2%) |
| P1 | PR | 1 (2%) | 4 (8%) |
| P1 | PD | 24 (49%) | 24 (49%) |
| P1 | SD | 22 (45%) | 5 (10%) |
| P1 | Unknown | 1 (2%) | 15 (31%) |
| P2 | CR | 1 (2%) | 1 (2%) |
| P2 | PR | 1 (2%) | 4 (8%) |
| P2 | PD | 24 (49%) | 25 (51%) |
| P2 | SD | 23 (47%) | 4 (8%) |
| P2 | Unknown | 0 | 15 (31%) |
| P3 | CR | 1 (2%) | 0 |
| P3 | PR | 2 (4%) | 6 (12%) |
| P3 | PD | 14 (29%) | 22 (45% |
| P3 | SD | 31 (63%) | 5 (10%) |
| P3 | Unknown | 1 (2%) | 16 (33%) |

**Figure S1:** Bar graph depicting the significant differences in the time needed for pre-operative and post-operative MRI volumetric segmentation of HGG via Neosoma HGG and manual segmentation software used by three investigators.

'*' indicates a statistically significant difference (p < 0.05).

**
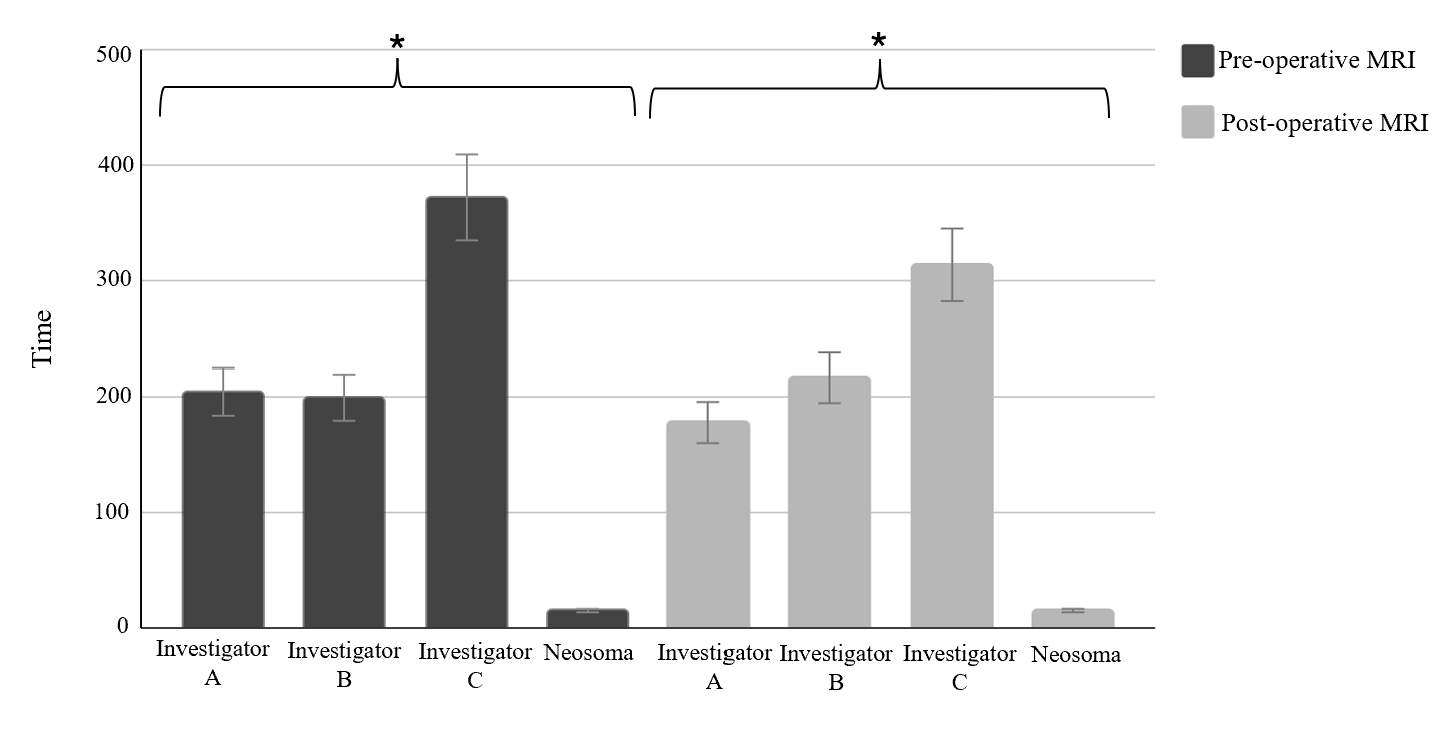
**
