# Supplementary figures and images for: Artificial intelligence-based volumetric measurements for longitudinal clinical assessment of treatment response in high-grade gliomas: Validation across institutional and public datasets
Source: Neurooncol Adv. 2026 Feb 17;8(1):vdag045. doi: 10.1093/noajnl/vdag045 (PMC12989098; doi:10.1093/noajnl/vdag045)

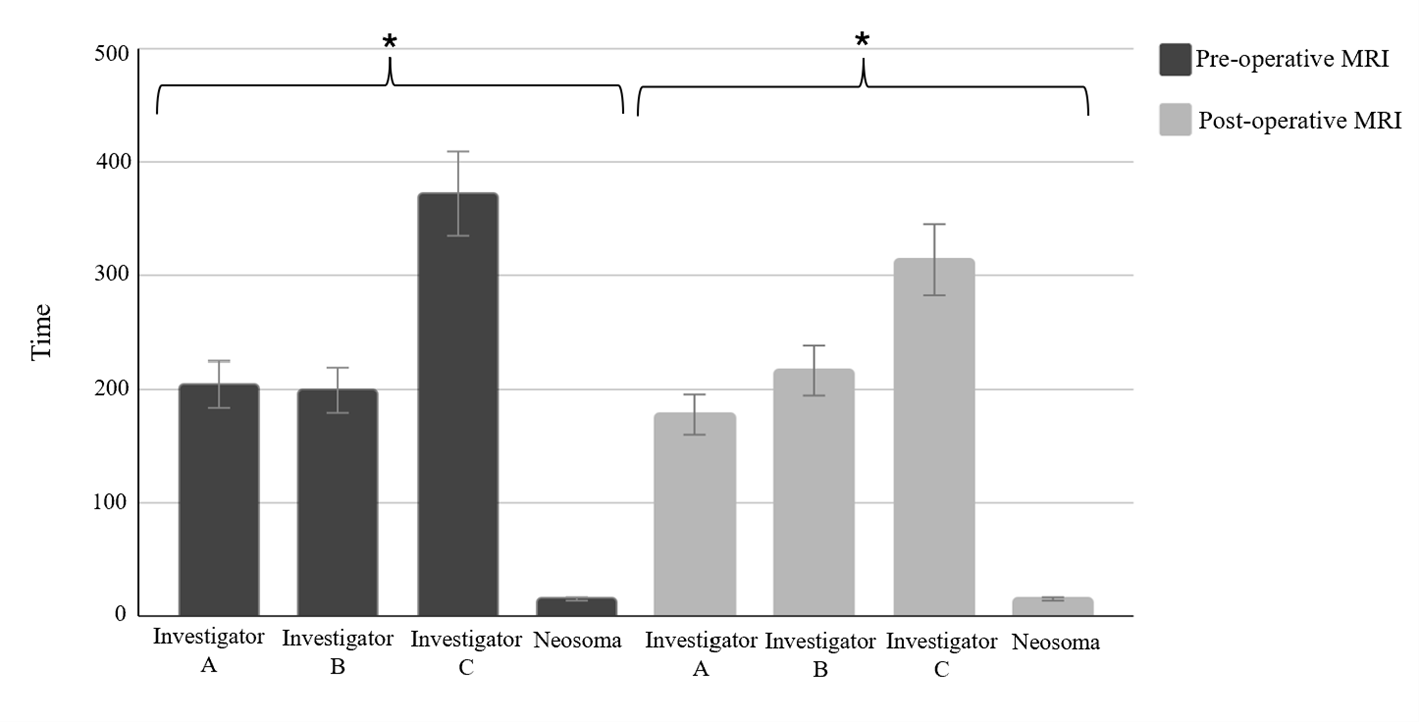

Supplement: vdag045_Supplementary_Data [file vdag045_supplementary_data.zip › Figure S1.tif]
